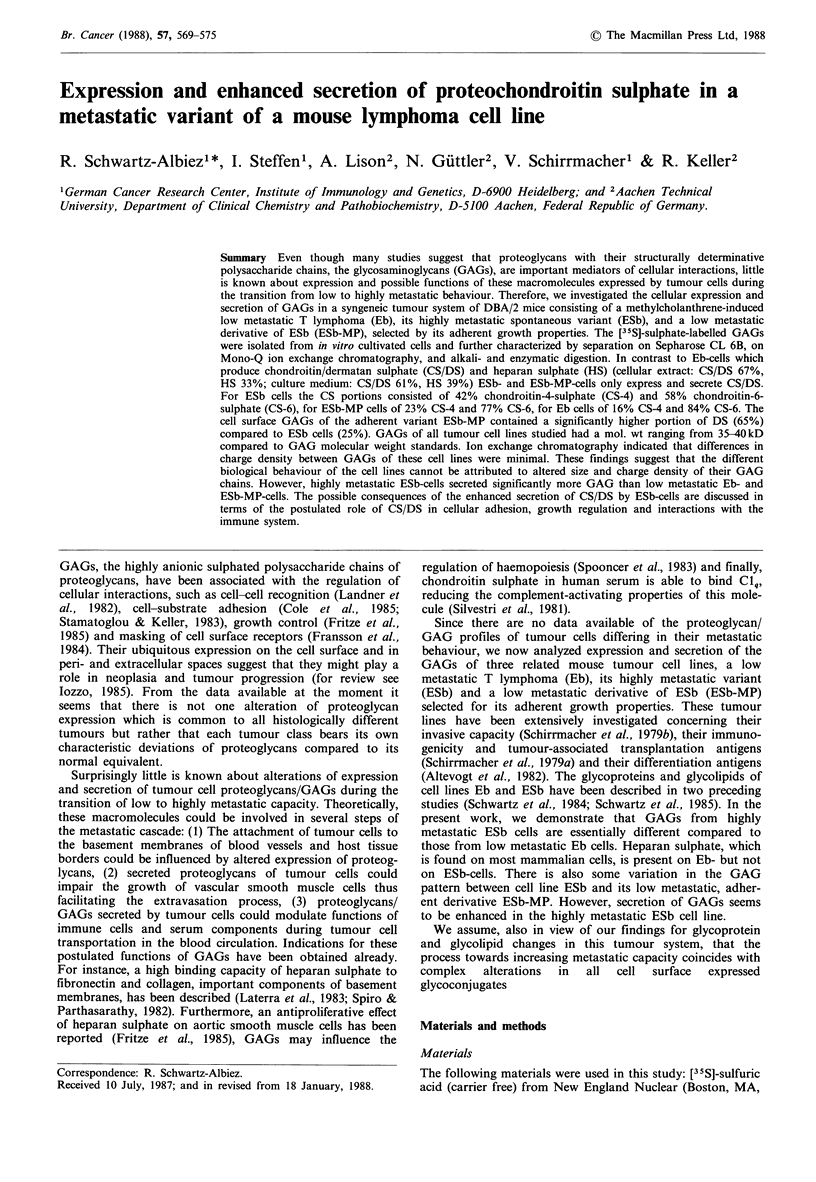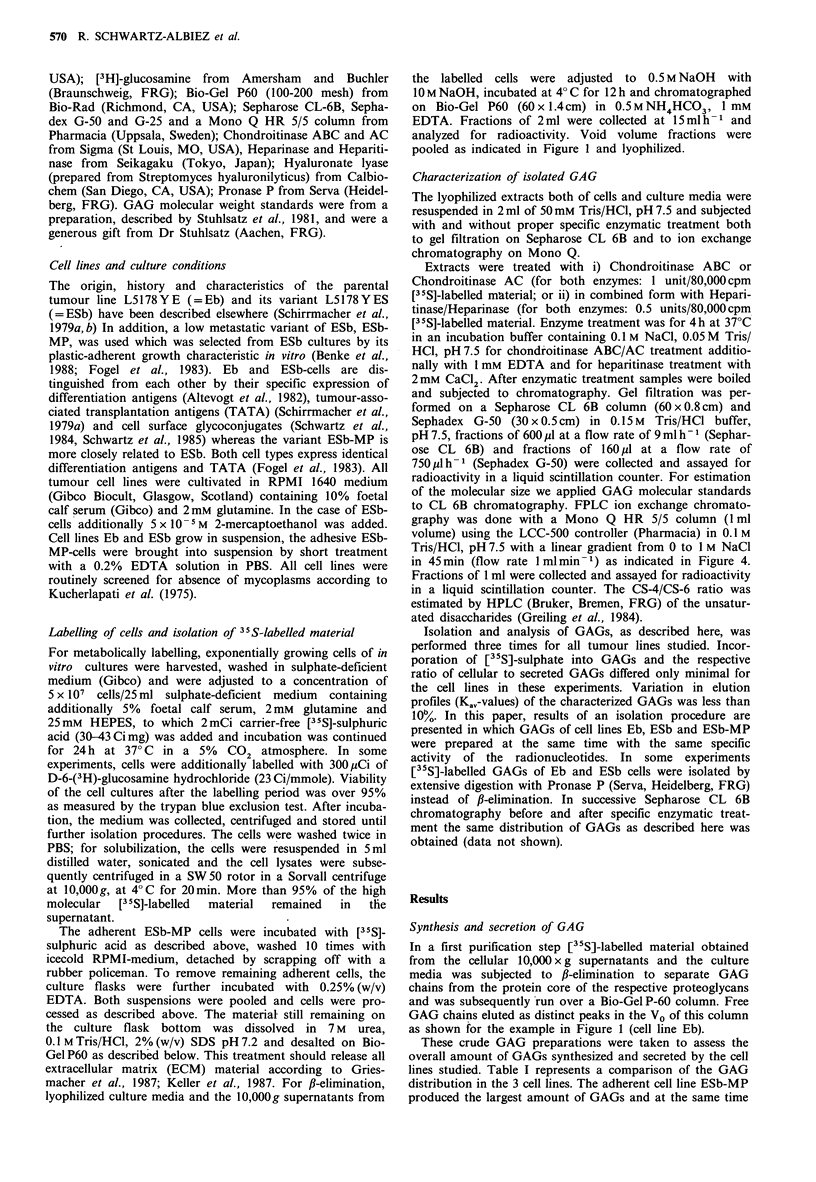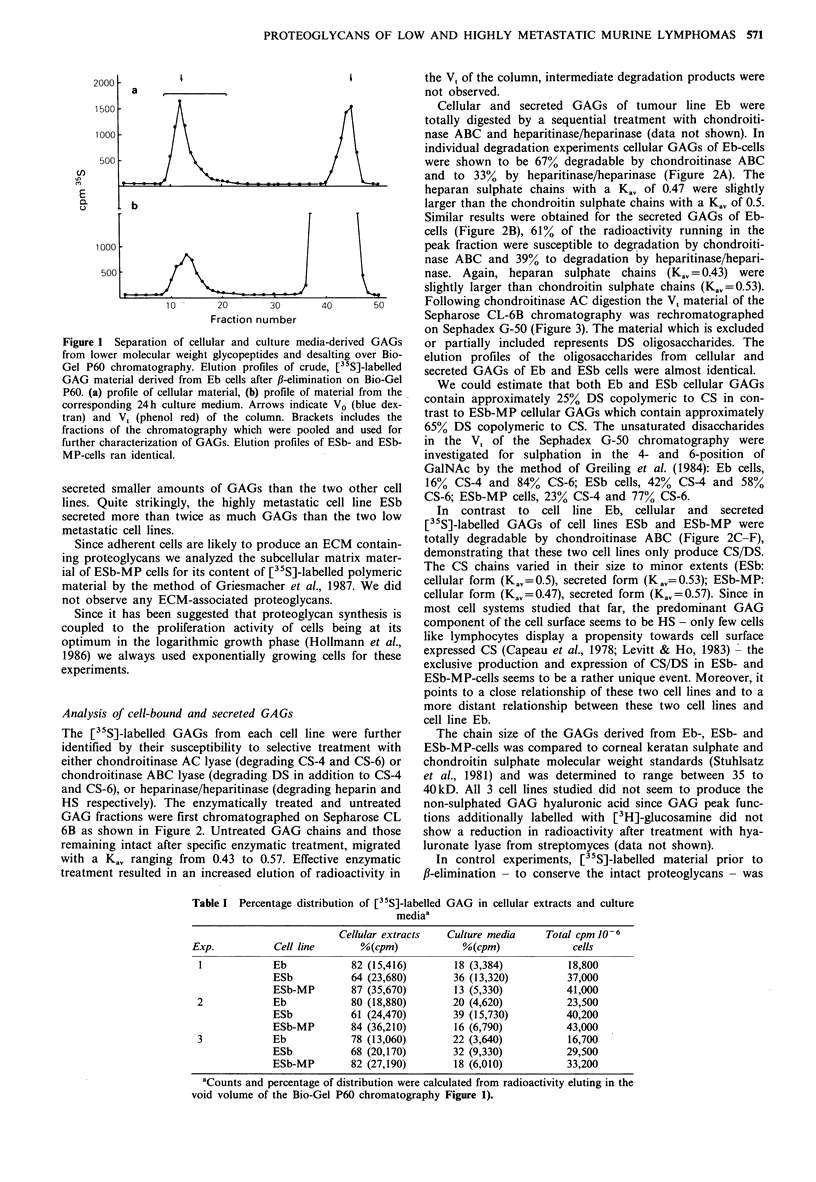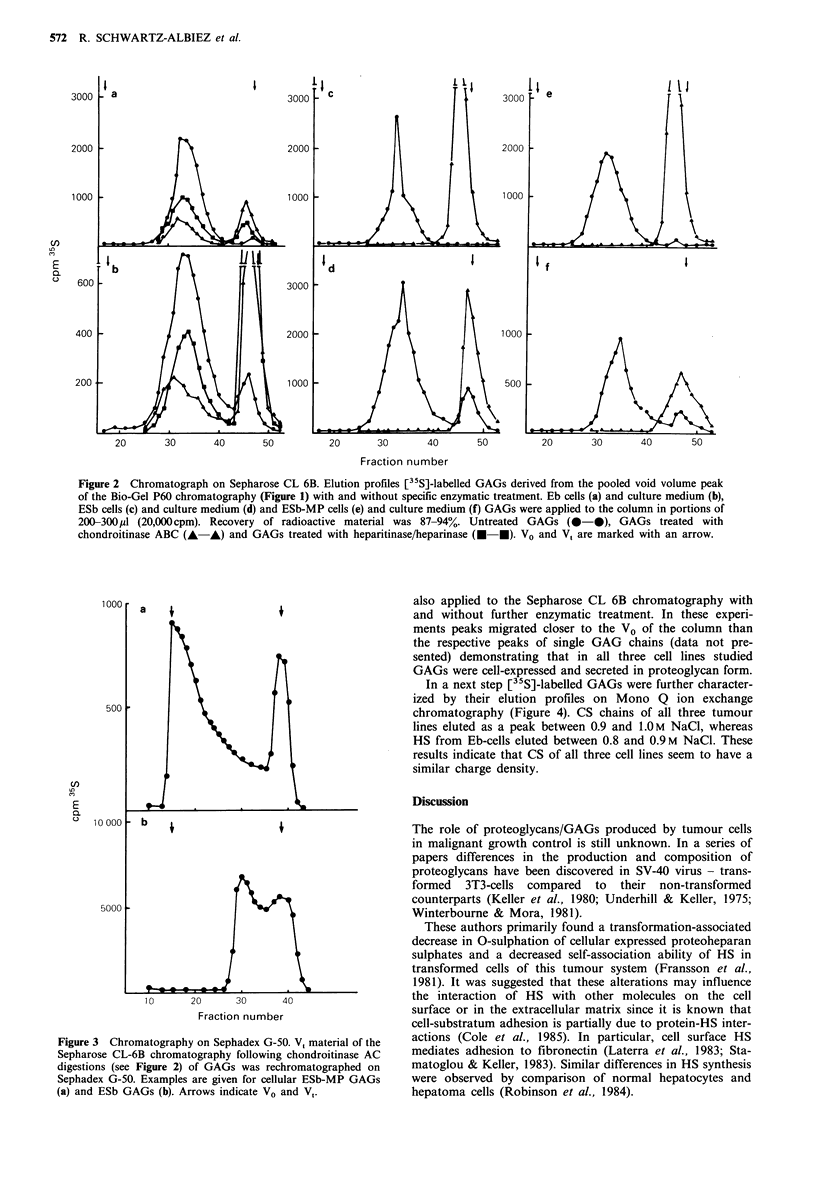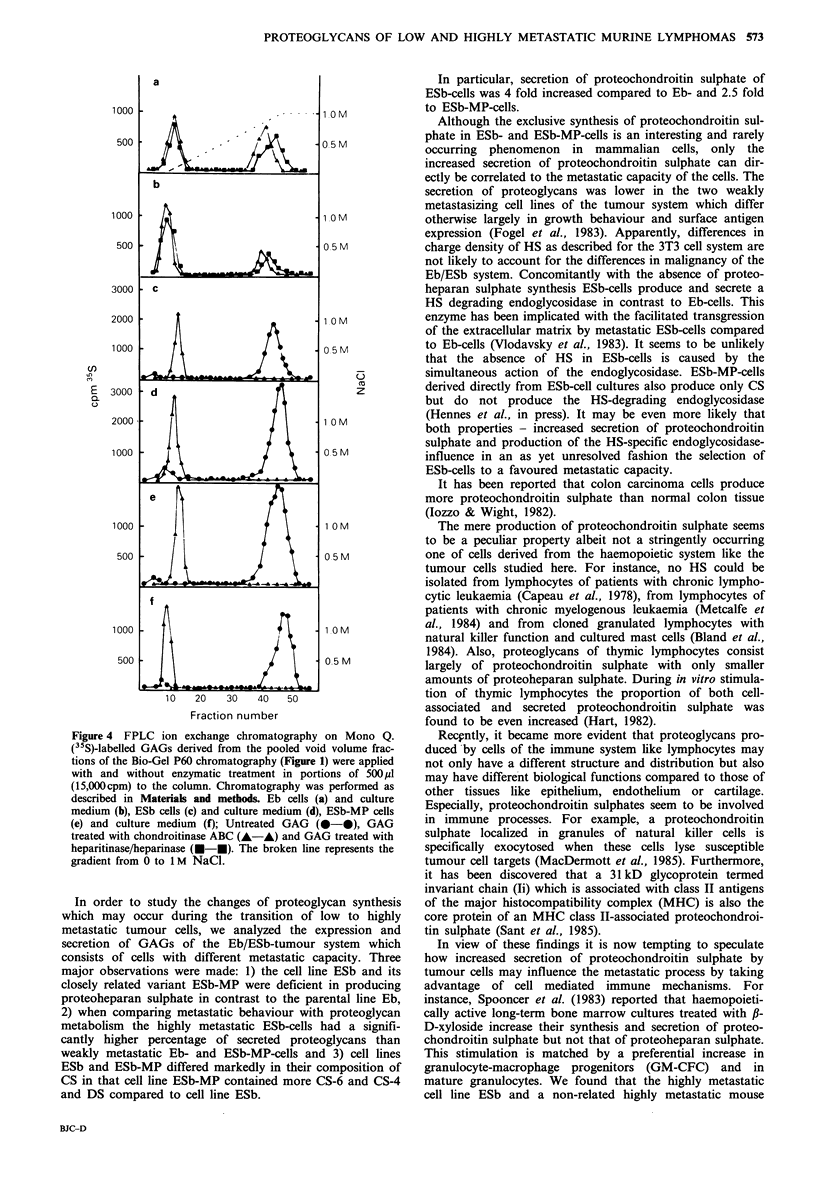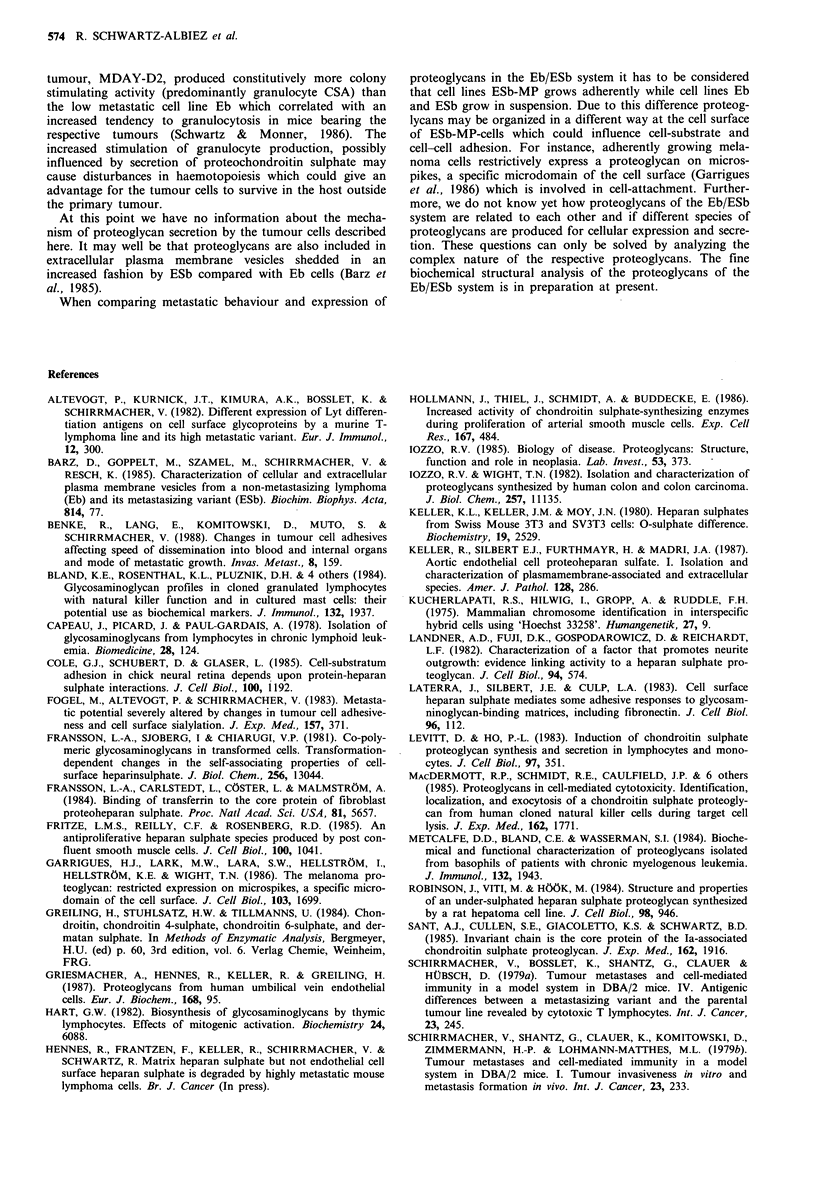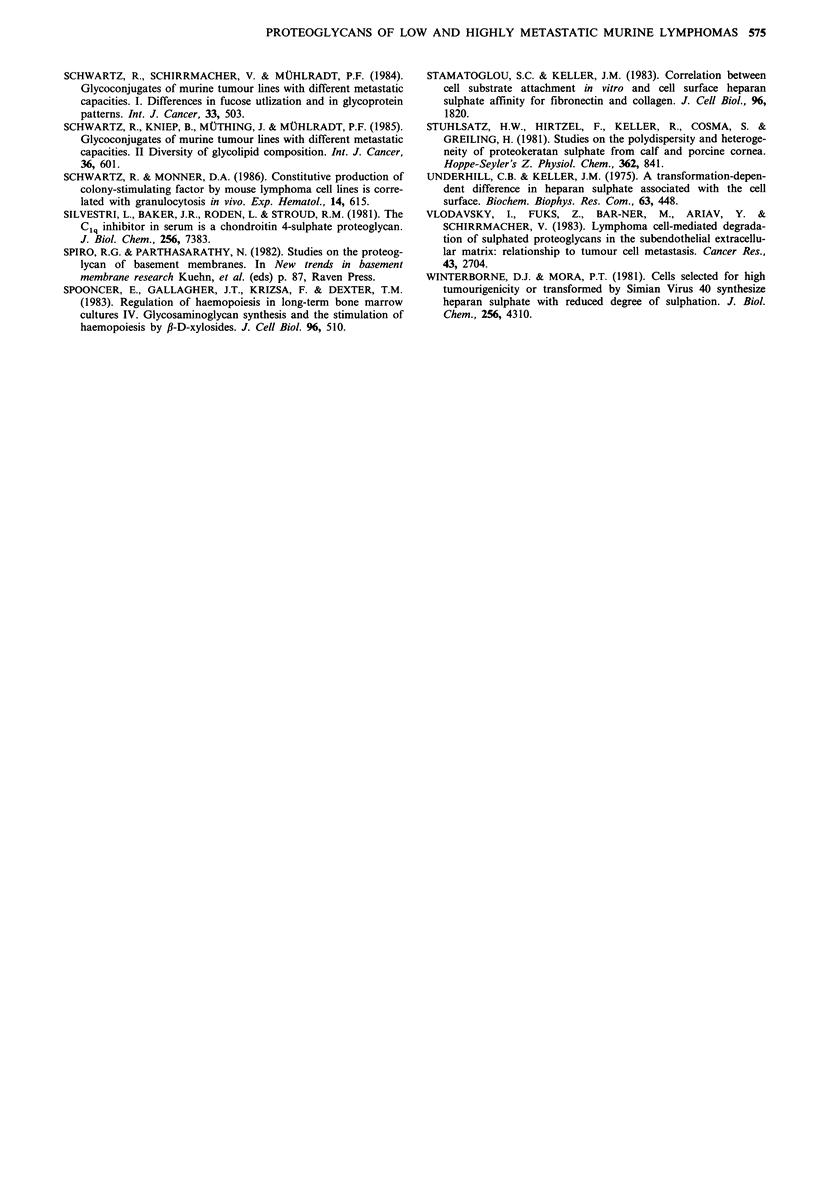# Expression and enhanced secretion of proteochondroitin sulphate in a metastatic variant of a mouse lymphoma cell line.

**DOI:** 10.1038/bjc.1988.130

**Published:** 1988-06

**Authors:** R. Schwartz-Albiez, I. Steffen, A. Lison, N. GÃ¼ttler, V. Schirrmacher, R. Keller

**Affiliations:** German Cancer Research Center, Institute of Immunology and Genetics, Heidelberg, Federal Republic of Germany.

## Abstract

Even though many studies suggest that proteoglycans with their structurally determinative polysaccharide chains, the glycosaminoglycans (GAGs), are important mediators of cellular interactions, little is known about expression and possible functions of these macromolecules expressed by tumour cells during the transition from low to highly metastatic behaviour. Therefore, we investigated the cellular expression and secretion of GAGs in a syngeneic tumour system of DBA/2 mice consisting of a methylcholanthrene-induced low metastatic T lymphoma (Eb), its highly metastatic spontaneous variant (ESb), and a low metastatic derivative of ESb (ESb-MP), selected by its adherent growth properties. The [35S]-sulphate-labelled GAGs were isolated from in vitro cultivated cells and further characterized by separation on Sepharose CL 6B, on Mono-Q ion exchange chromatography, and alkali- and enzymatic digestion. In contrast to Eb-cells which produce chondroitin/dermatan sulphate (CS/DS) and heparan sulphate (HS) (cellular extract: CS/DS 67%, HS 33%; culture medium: CS/DS 61%, HS 39%) ESb- and ESb-MP-cells only express and secrete CS/DS. For ESb cells the CS portions consisted of 42% chondroitin-4-sulphate (CS-4) and 58% chondroitin-6-sulphate (CS-6), for ESb-MP cells of 23% CS-4 and 77% CS-6, for Eb cells of 16% CS-4 and 84% CS-6. The cell surface GAGs of the adherent variant ESb-MP contained a significantly higher portion of DS (65%) compared to ESb cells (25%). GAGs of all tumour cell lines studied had a mol. wt ranging from 35-40 kD compared to GAG molecular weight standards. Ion exchange chromatography indicated that differences in charge density between GAGs of these cell lines were minimal. These findings suggest that the different biological behaviour of the cell lines cannot be attributed to altered size and charge density of their GAG chains. However, highly metastatic ESb-cells secreted significantly more GAG than low metastatic Eb- and ESb-MP-cells. The possible consequences of the enhanced secretion of CS/DS by ESb-cells are discussed in terms of the postulated role of CS/DS in cellular adhesion, growth regulation and interactions with the immune system.


Br. Cancer (1988), 57, 569–575 © The Macmillan Press Ltd, 1988

### Expression and enhanced secretion of proteochondroitin sulphate in a metastatic variant of a mouse lymphoma cell line

R. Schwartz-Albiez[1]*, I. Steffen[1], A. Lison[2], N. Güttler[2], V. Schirrmacher[1] & R. Keller[2]

[1]*German Cancer Research Center, Institute of Immunology and Genetics, D-6900 Heidelberg; and* [2]*Aachen Technical University, Department of Clinical Chemistry and Pathobiochemistry, D-5100 Aachen, Federal Republic of Germany.*

**Summary** Even though many studies suggest that proteoglycans with their structurally determinative polysaccharide chains, the glycosaminoglycans (GAGs), are important mediators of cellular interactions, little is known about expression and possible functions of these macromolecules expressed by tumour cells during the transition from low to highly metastatic behaviour. Therefore, we investigated the cellular expression and secretion of GAGs in a syngeneic tumour system of DBA/2 mice consisting of a methylcholanthrene-induced low metastatic T lymphoma (Eb), its highly metastatic spontaneous variant (ESb), and a low metastatic derivative of ESb (ESb-MP), selected by its adherent growth properties. The [$^{35}$S]-sulphate-labelled GAGs were isolated from *in vitro* cultivated cells and further characterized by separation on Sepharose CL 6B, on Mono-Q ion exchange chromatography, and alkali- and enzymatic digestion. In contrast to Eb-cells which produce chondroitin/dermatan sulphate (CS/DS) and heparan sulphate (HS) (cellular extract: CS/DS 67%, HS 33%; culture medium: CS/DS 61%, HS 39%) ESb- and ESb-MP-cells only express and secrete CS/DS. For ESb cells the CS portions consisted of 42% chondroitin-4-sulphate (CS-4) and 58% chondroitin-6-sulphate (CS-6), for ESb-MP cells of 23% CS-4 and 77% CS-6, for Eb cells of 16% CS-4 and 84% CS-6. The cell surface GAGs of the adherent variant ESb-MP contained a significantly higher portion of DS (65%) compared to ESb cells (25%). GAGs of all tumour cell lines studied had a mol. wt ranging from 35–40 kD compared to GAG molecular weight standards. Ion exchange chromatography indicated that differences in charge density between GAGs of these cell lines were minimal. These findings suggest that the different biological behaviour of the cell lines cannot be attributed to altered size and charge density of their GAG chains. However, highly metastatic ESb-cells secreted significantly more GAG than low metastatic Eb- and ESb-MP-cells. The possible consequences of the enhanced secretion of CS/DS by ESb-cells are discussed in terms of the postulated role of CS/DS in cellular adhesion, growth regulation and interactions with the immune system.

GAGs, the highly anionic sulphated polysaccharide chains of proteoglycans, have been associated with the regulation of cellular interactions, such as cell–cell recognition (Landner *et al.*, 1982), cell–substrate adhesion (Cole *et al.*, 1985; Stamatoglou & Keller, 1983), growth control (Fritze *et al.*, 1985) and masking of cell surface receptors (Fransson *et al.*, 1984). Their ubiquitous expression on the cell surface and in peri- and extracellular spaces suggest that they might play a role in neoplasia and tumour progression (for review see Iozzo, 1985). From the data available at the moment it seems that there is not one alteration of proteoglycan expression which is common to all histologically different tumours but rather that each tumour class bears its own characteristic deviations of proteoglycans compared to its normal equivalent.

Surprisingly little is known about alterations of expression and secretion of tumour cell proteoglycans/GAGs during the transition of low to highly metastatic capacity. Theoretically, these macromolecules could be involved in several steps of the metastatic cascade: (1) The attachment of tumour cells to the basement membranes of blood vessels and host tissue borders could be influenced by altered expression of proteoglycans, (2) secreted proteoglycans of tumour cells could impair the growth of vascular smooth muscle cells thus facilitating the extravasation process, (3) proteoglycans/GAGs secreted by tumour cells could modulate functions of immune cells and serum components during tumour cell transportation in the blood circulation. Indications for these postulated functions of GAGs have been obtained already. For instance, a high binding capacity of heparan sulphate to fibronectin and collagen, important components of basement membranes, has been described (Laterra *et al.*, 1983; Spiro & Parthasarathy, 1982). Furthermore, an antiproliferative effect of heparan sulphate on aortic smooth muscle cells has been reported (Fritze *et al.*, 1985), GAGs may influence the regulation of haemopoiesis (Spooncer *et al.*, 1983) and finally, chondroitin sulphate in human serum is able to bind $C1_q$, reducing the complement-activating properties of this molecule (Silvestri *et al.*, 1981).

Since there are no data available of the proteoglycan/GAG profiles of tumour cells differing in their metastatic behaviour, we now analyzed expression and secretion of the GAGs of three related mouse tumour cell lines, a low metastatic T lymphoma (Eb), its highly metastatic variant (ESb) and a low metastatic derivative of ESb (ESb-MP) selected for its adherent growth properties. These tumour lines have been extensively investigated concerning their invasive capacity (Schirrmacher *et al.*, 1979*b*), their immunogenicity and tumour-associated transplantation antigens (Schirrmacher *et al.*, 1979*a*) and their differentiation antigens (Altevogt *et al.*, 1982). The glycoproteins and glycolipids of cell lines Eb and ESb have been described in two preceding studies (Schwartz *et al.*, 1984; Schwartz *et al.*, 1985). In the present work, we demonstrate that GAGs from highly metastatic ESb cells are essentially different compared to those from low metastatic Eb cells. Heparan sulphate, which is found on most mammalian cells, is present on Eb- but not on ESb-cells. There is also some variation in the GAG pattern between cell line ESb and its low metastatic, adherent derivative ESb-MP. However, secretion of GAGs seems to be enhanced in the highly metastatic ESb cell line.

We assume, also in view of our findings for glycoprotein and glycolipid changes in this tumour system, that the process towards increasing metastatic capacity coincides with complex alterations in all cell surface expressed glycoconjugates

### Materials and methods

### *Materials*

The following materials were used in this study: [$^{35}$S]-sulfuric acid (carrier free) from New England Nuclear (Boston, MA,

Correspondence: R. Schwartz-Albiez.
Received 10 July, 1987; and in revised from 18 January, 1988.

USA); [$^3$H]-glucosamine from Amersham and Buchler (Braunschweig, FRG); Bio-Gel P60 (100-200 mesh) from Bio-Rad (Richmond, CA, USA); Sepharose CL-6B, Sephadex G-50 and G-25 and a Mono Q HR 5/5 column from Pharmacia (Uppsala, Sweden); Chondroitinase ABC and AC from Sigma (St Louis, MO, USA), Heparinase and Heparitinase from Seikagaku (Tokyo, Japan); Hyaluronate lyase (prepared from Streptomyces hyaluronilyticus) from Calbiochem (San Diego, CA, USA); Pronase P from Serva (Heidelberg, FRG). GAG molecular weight standards were from a preparation, described by Stuhlsatz *et al.*, 1981, and were a generous gift from Dr Stuhlsatz (Aachen, FRG).

*Cell lines and culture conditions*

The origin, history and characteristics of the parental tumour line L5178Y E (=Eb) and its variant L5178Y ES (=ESb) have been described elsewhere (Schirrmacher *et al.*, 1979*a, b*) In addition, a low metastatic variant of ESb, ESb-MP, was used which was selected from ESb cultures by its plastic-adherent growth characteristic *in vitro* (Benke *et al.*, 1988; Fogel *et al.*, 1983). Eb and ESb-cells are distinguished from each other by their specific expression of differentiation antigens (Altevogt *et al.*, 1982), tumour-associated transplantation antigens (TATA) (Schirrmacher *et al.*, 1979*a*) and cell surface glycoconjugates (Schwartz *et al.*, 1984, Schwartz *et al.*, 1985) whereas the variant ESb-MP is more closely related to ESb. Both cell types express identical differentiation antigens and TATA (Fogel *et al.*, 1983). All tumour cell lines were cultivated in RPMI 1640 medium (Gibco Biocult, Glasgow, Scotland) containing 10% foetal calf serum (Gibco) and 2 mM glutamine. In the case of ESb-cells additionally $5\times10^{-5}$ M 2-mercaptoethanol was added. Cell lines Eb and ESb grow in suspension, the adhesive ESb-MP-cells were brought into suspension by short treatment with a 0.2% EDTA solution in PBS. All cell lines were routinely screened for absence of mycoplasms according to Kucherlapati *et al.* (1975).

*Labelling of cells and isolation of $^{35}$S-labelled material*

For metabolically labelling, exponentially growing cells of *in vitro* cultures were harvested, washed in sulphate-deficient medium (Gibco) and were adjusted to a concentration of $5\times10^7$ cells/25 ml sulphate-deficient medium containing additionally 5% foetal calf serum, 2 mM glutamine and 25 mM HEPES, to which 2 mCi carrier-free [$^{35}$S]-sulphuric acid (30–43 Ci mg) was added and incubation was continued for 24 h at 37°C in a 5% $CO_2$ atmosphere. In some experiments, cells were additionally labelled with 300 $\mu$Ci of D-6-($^3$H)-glucosamine hydrochloride (23 Ci/mmole). Viability of the cell cultures after the labelling period was over 95% as measured by the trypan blue exclusion test. After incubation, the medium was collected, centrifuged and stored until further isolation procedures. The cells were washed twice in PBS; for solubilization, the cells were resuspended in 5 ml distilled water, sonicated and the cell lysates were subsequently centrifuged in a SW 50 rotor in a Sorvall centrifuge at 10,000 *g*, at 4°C for 20 min. More than 95% of the high molecular [$^{35}$S]-labelled material remained in the supernatant.

The adherent ESb-MP cells were incubated with [$^{35}$S]-sulphuric acid as described above, washed 10 times with icecold RPMI-medium, detached by scrapping off with a rubber policeman. To remove remaining adherent cells, the culture flasks were further incubated with 0.25% (w/v) EDTA. Both suspensions were pooled and cells were processed as described above. The material still remaining on the culture flask bottom was dissolved in 7 M urea, 0.1 M Tris/HCl, 2% (w/v) SDS pH 7.2 and desalted on Bio-Gel P60 as described below. This treatment should release all extracellular matrix (ECM) material according to Griesmacher *et al.*, 1987; Keller *et al.*, 1987. For $\beta$-elimination, lyophilized culture media and the 10,000 *g* supernatants from the labelled cells were adjusted to 0.5 M NaOH with 10 M NaOH, incubated at 4°C for 12 h and chromatographed on Bio-Gel P60 (60 × 1.4 cm) in 0.5 M $NH_4HCO_3$, 1 mM EDTA. Fractions of 2 ml were collected at 15 ml h$^{-1}$ and analyzed for radioactivity. Void volume fractions were pooled as indicated in Figure 1 and lyophilized.

*Characterization of isolated GAG*

The lyophilized extracts both of cells and culture media were resuspended in 2 ml of 50 mM Tris/HCl, pH 7.5 and subjected with and without proper specific enzymatic treatment both to gel filtration on Sepharose CL 6B and to ion exchange chromatography on Mono Q.

Extracts were treated with i) Chondroitinase ABC or Chondroitinase AC (for both enzymes: 1 unit/80,000 cpm [$^{35}$S]-labelled material; or ii) in combined form with Heparitinase/Heparinase (for both enzymes: 0.5 units/80,000 cpm [$^{35}$S]-labelled material. Enzyme treatment was for 4 h at 37°C in an incubation buffer containing 0.1 M NaCl, 0.05 M Tris/HCl, pH 7.5 for chondroitinase ABC/AC treatment additionally with 1 mM EDTA and for heparitinase treatment with 2 mM $CaCl_2$. After enzymatic treatment samples were boiled and subjected to chromatography. Gel filtration was performed on a Sepharose CL 6B column (60 × 0.8 cm) and Sephadex G-50 (30 × 0.5 cm) in 0.15 M Tris/HCl buffer, pH 7.5, fractions of 600 $\mu$l at a flow rate of 9 ml h$^{-1}$ (Sepharose CL 6B) and fractions of 160 $\mu$l at a flow rate of 750 $\mu$l h$^{-1}$ (Sephadex G-50) were collected and assayed for radioactivity in a liquid scintillation counter. For estimation of the molecular size we applied GAG molecular standards to CL 6B chromatography. FPLC ion exchange chromatography was done with a Mono Q HR 5/5 column (1 ml volume) using the LCC-500 controller (Pharmacia) in 0.1 M Tris/HCl, pH 7.5 with a linear gradient from 0 to 1 M NaCl in 45 min (flow rate 1 ml min$^{-1}$) as indicated in Figure 4. Fractions of 1 ml were collected and assayed for radioactivity in a liquid scintillation counter. The CS-4/CS-6 ratio was estimated by HPLC (Bruker, Bremen, FRG) of the unsaturated disaccharides (Greiling *et al.*, 1984).

Isolation and analysis of GAGs, as described here, was performed three times for all tumour lines studied. Incorporation of [$^{35}$S]-sulphate into GAGs and the respective ratio of cellular to secreted GAGs differed only minimal for the cell lines in these experiments. Variation in elution profiles ($K_{av}$-values) of the characterized GAGs was less than 10%. In this paper, results of an isolation procedure are presented in which GAGs of cell lines Eb, ESb and ESb-MP were prepared at the same time with the same specific activity of the radionucleotides. In some experiments [$^{35}$S]-labelled GAGs of Eb and ESb cells were isolated by extensive digestion with Pronase P (Serva, Heidelberg, FRG) instead of $\beta$-elimination. In successive Sepharose CL 6B chromatography before and after specific enzymatic treatment the same distribution of GAGs as described here was obtained (data not shown).

### Results

*Synthesis and secretion of GAG*

In a first purification step [$^{35}$S]-labelled material obtained from the cellular 10,000 × g supernatants and the culture media was subjected to $\beta$-elimination to separate GAG chains from the protein core of the respective proteoglycans and was subsequently run over a Bio-Gel P-60 column. Free GAG chains eluted as distinct peaks in the $V_0$ of this column as shown for the example in Figure 1 (cell line Eb).

These crude GAG preparations were taken to assess the overall amount of GAGs synthesized and secreted by the cell lines studied. Table I represents a comparison of the GAG distribution in the 3 cell lines. The adherent cell line ESb-MP produced the largest amount of GAGs and at the same time

**Figure 1** Separation of cellular and culture media-derived GAGs from lower molecular weight glycopeptides and desalting over Bio-Gel P60 chromatography. Elution profiles of crude, [$^{35}$S]-labelled GAG material derived from Eb cells after $\beta$-elimination on Bio-Gel P60. (**a**) profile of cellular material, (**b**) profile of material from the corresponding 24 h culture medium. Arrows indicate $V_0$ (blue dextran) and $V_t$ (phenol red) of the column. Brackets includes the fractions of the chromatography which were pooled and used for further characterization of GAGs. Elution profiles of ESb- and ESb-MP-cells ran identical.

secreted smaller amounts of GAGs than the two other cell lines. Quite strikingly, the highly metastatic cell line ESb secreted more than twice as much GAGs than the two low metastatic cell lines.

Since adherent cells are likely to produce an ECM containing proteoglycans we analyzed the subcellular matrix material of ESb-MP cells for its content of [$^{35}$S]-labelled polymeric material by the method of Griesmacher *et al.*, 1987. We did not observe any ECM-associated proteoglycans.

Since it has been suggested that proteoglycan synthesis is coupled to the proliferation activity of cells being at its optimum in the logarithmic growth phase (Hollmann *et al.*, 1986) we always used exponentially growing cells for these experiments.

*Analysis of cell-bound and secreted GAGs*

The [$^{35}$S]-labelled GAGs from each cell line were further identified by their susceptibility to selective treatment with either chondroitinase AC lyase (degrading CS-4 and CS-6) or chondroitinase ABC lyase (degrading DS in addition to CS-4 and CS-6), or heparinase/heparitinase (degrading heparin and HS respectively). The enzymatically treated and untreated GAG fractions were first chromatographed on Sepharose CL 6B as shown in Figure 2. Untreated GAG chains and those remaining intact after specific enzymatic treatment, migrated with a $K_{av}$ ranging from 0.43 to 0.57. Effective enzymatic treatment resulted in an increased elution of radioactivity in the $V_t$ of the column, intermediate degradation products were not observed.

Cellular and secreted GAGs of tumour line Eb were totally digested by a sequential treatment with chondroitinase ABC and heparitinase/heparinase (data not shown). In individual degradation experiments cellular GAGs of Eb-cells were shown to be 67% degradable by chondroitinase ABC and to 33% by heparitinase/heparinase (Figure 2A). The heparan sulphate chains with a $K_{av}$ of 0.47 were slightly larger than the chondroitin sulphate chains with a $K_{av}$ of 0.5. Similar results were obtained for the secreted GAGs of Eb-cells (Figure 2B), 61% of the radioactivity running in the peak fraction were susceptible to degradation by chondroitinase ABC and 39% to degradation by heparitinase/heparinase. Again, heparan sulphate chains ($K_{av}$=0.43) were slightly larger than chondroitin sulphate chains ($K_{av}$=0.53). Following chondroitinase AC digestion the $V_t$ material of the Sepharose CL-6B chromatography was rechromatographed on Sephadex G-50 (Figure 3). The material which is excluded or partially included represents DS oligosaccharides. The elution profiles of the oligosaccharides from cellular and secreted GAGs of Eb and ESb cells were almost identical.

We could estimate that both Eb and ESb cellular GAGs contain approximately 25% DS copolymeric to CS in contrast to ESb-MP cellular GAGs which contain approximately 65% DS copolymeric to CS. The unsaturated disaccharides in the $V_t$ of the Sephadex G-50 chromatography were investigated for sulphation in the 4- and 6-position of GalNAc by the method of Greiling *et al.* (1984): Eb cells, 16% CS-4 and 84% CS-6; ESb cells, 42% CS-4 and 58% CS-6; ESb-MP cells, 23% CS-4 and 77% CS-6.

In contrast to cell line Eb, cellular and secreted [$^{35}$S]-labelled GAGs of cell lines ESb and ESb-MP were totally degradable by chondroitinase ABC (Figure 2C–F), demonstrating that these two cell lines only produce CS/DS. The CS chains varied in their size to minor extents (ESb: cellular form ($K_{av}$=0.5), secreted form ($K_{av}$=0.53); ESb-MP: cellular form ($K_{av}$=0.47), secreted form ($K_{av}$=0.57). Since in most cell systems studied that far, the predominant GAG component of the cell surface seems to be HS – only few cells like lymphocytes display a propensity towards cell surface expressed CS (Capeau *et al.*, 1978; Levitt & Ho, 1983) – the exclusive production and expression of CS/DS in ESb- and ESb-MP-cells seems to be a rather unique event. Moreover, it points to a close relationship of these two cell lines and to a more distant relationship between these two cell lines and cell line Eb.

The chain size of the GAGs derived from Eb-, ESb- and ESb-MP-cells was compared to corneal keratan sulphate and chondroitin sulphate molecular weight standards (Stuhlsatz *et al.*, 1981) and was determined to range between 35 to 40 kD. All 3 cell lines studied did not seem to produce the non-sulphated GAG hyaluronic acid since GAG peak functions additionally labelled with [$^{3}$H]-glucosamine did not show a reduction in radioactivity after treatment with hyaluronate lyase from streptomyces (data not shown).

In control experiments, [$^{35}$S]-labelled material prior to $\beta$-elimination – to conserve the intact proteoglycans – was

**Table I** Percentage distribution of [$^{35}$S]-labelled GAG in cellular extracts and culture media[a]

| *Exp.* | *Cell line* | *Cellular extracts %(cpm)* | *Culture media %(cpm)* | *Total cpm $10^{-6}$ cells* |
|---|---|---|---|---|
| 1 | Eb | 82 (15,416) | 18 (3,384) | 18,800 |
| | ESb | 64 (23,680) | 36 (13,320) | 37,000 |
| | ESb-MP | 87 (35,670) | 13 (5,330) | 41,000 |
| 2 | Eb | 80 (18,880) | 20 (4,620) | 23,500 |
| | ESb | 61 (24,470) | 39 (15,730) | 40,200 |
| | ESb-MP | 84 (36,210) | 16 (6,790) | 43,000 |
| 3 | Eb | 78 (13,060) | 22 (3,640) | 16,700 |
| | ESb | 68 (20,170) | 32 (9,330) | 29,500 |
| | ESb-MP | 82 (27,190) | 18 (6,010) | 33,200 |

[a]Counts and percentage of distribution were calculated from radioactivity eluting in the void volume of the Bio-Gel P60 chromatography **Figure 1**).

**Figure 2** Chromatograph on Sepharose CL 6B. Elution profiles [$^{35}$S]-labelled GAGs derived from the pooled void volume peak of the Bio-Gel P60 chromatography (**Figure 1**) with and without specific enzymatic treatment. Eb cells (**a**) and culture medium (**b**), ESb cells (**c**) and culture medium (**d**) and ESb-MP cells (**e**) and culture medium (**f**) GAGs were applied to the column in portions of 200–300 μl (20,000 cpm). Recovery of radioactive material was 87–94%. Untreated GAGs (●—●), GAGs treated with chondroitinase ABC (▲—▲) and GAGs treated with heparitinase/heparinase (■—■). $V_0$ and $V_t$ are marked with an arrow.

**Figure 3** Chromatography on Sephadex G-50. $V_t$ material of the Sepharose CL-6B chromatography following chondroitinase AC digestions (see **Figure 2**) of GAGs was rechromatographed on Sephadex G-50. Examples are given for cellular ESb-MP GAGs (**a**) and ESb GAGs (**b**). Arrows indicate $V_0$ and $V_t$.

also applied to the Sepharose CL 6B chromatography with and without further enzymatic treatment. In these experiments peaks migrated closer to the $V_0$ of the column than the respective peaks of single GAG chains (data not presented) demonstrating that in all three cell lines studied GAGs were cell-expressed and secreted in proteoglycan form.

In a next step [$^{35}$S]-labelled GAGs were further characterized by their elution profiles on Mono Q ion exchange chromatography (Figure 4). CS chains of all three tumour lines eluted as a peak between 0.9 and 1.0 M NaCl, whereas HS from Eb-cells eluted between 0.8 and 0.9 M NaCl. These results indicate that CS of all three cell lines seem to have a similar charge density.

### Discussion

The role of proteoglycans/GAGs produced by tumour cells in malignant growth control is still unknown. In a series of papers differences in the production and composition of proteoglycans have been discovered in SV-40 virus – transformed 3T3-cells compared to their non-transformed counterparts (Keller *et al.*, 1980; Underhill & Keller, 1975; Winterbourne & Mora, 1981).

These authors primarily found a transformation-associated decrease in O-sulphation of cellular expressed proteoheparan sulphates and a decreased self-association ability of HS in transformed cells of this tumour system (Fransson *et al.*, 1981). It was suggested that these alterations may influence the interaction of HS with other molecules on the cell surface or in the extracellular matrix since it is known that cell-substratum adhesion is partially due to protein-HS interactions (Cole *et al.*, 1985). In particular, cell surface HS mediates adhesion to fibronectin (Laterra *et al.*, 1983; Stamatoglou & Keller, 1983). Similar differences in HS synthesis were observed by comparison of normal hepatocytes and hepatoma cells (Robinson *et al.*, 1984).

**Figure 4** FPLC ion exchange chromatography on Mono Q. ($^{35}$S)-labelled GAGs derived from the pooled void volume fractions of the Bio-Gel P60 chromatography (**Figure 1**) were applied with and without enzymatic treatment in portions of 500 $\mu$l (15,000 cpm) to the column. Chromatography was performed as described in **Materials and methods.** Eb cells (**a**) and culture medium (**b**), ESb cells (**c**) and culture medium (**d**), ESb-MP cells (**e**) and culture medium (**f**); Untreated GAG (●—●), GAG treated with chondroitinase ABC (▲—▲) and GAG treated with heparitinase/heparinase (■—■). The broken line represents the gradient from 0 to 1 M NaCl.

In order to study the changes of proteoglycan synthesis which may occur during the transition of low to highly metastatic tumour cells, we analyzed the expression and secretion of GAGs of the Eb/ESb-tumour system which consists of cells with different metastatic capacity. Three major observations were made: 1) the cell line ESb and its closely related variant ESb-MP were deficient in producing proteoheparan sulphate in contrast to the parental line Eb, 2) when comparing metastatic behaviour with proteoglycan metabolism the highly metastatic ESb-cells had a significantly higher percentage of secreted proteoglycans than weakly metastatic Eb- and ESb-MP-cells and 3) cell lines ESb and ESb-MP differed markedly in their composition of CS in that cell line ESb-MP contained more CS-6 and CS-4 and DS compared to cell line ESb.

In particular, secretion of proteochondroitin sulphate of ESb-cells was 4 fold increased compared to Eb- and 2.5 fold to ESb-MP-cells.

Although the exclusive synthesis of proteochondroitin sulphate in ESb- and ESb-MP-cells is an interesting and rarely occurring phenomenon in mammalian cells, only the increased secretion of proteochondroitin sulphate can directly be correlated to the metastatic capacity of the cells. The secretion of proteoglycans was lower in the two weakly metastasizing cell lines of the tumour system which differ otherwise largely in growth behaviour and surface antigen expression (Fogel *et al.*, 1983). Apparently, differences in charge density of HS as described for the 3T3 cell system are not likely to account for the differences in malignancy of the Eb/ESb system. Concomitantly with the absence of proteoheparan sulphate synthesis ESb-cells produce and secrete a HS degrading endoglycosidase in contrast to Eb-cells. This enzyme has been implicated with the facilitated transgression of the extracellular matrix by metastatic ESb-cells compared to Eb-cells (Vlodavsky *et al.*, 1983). It seems to be unlikely that the absence of HS in ESb-cells is caused by the simultaneous action of the endoglycosidase. ESb-MP-cells derived directly from ESb-cell cultures also produce only CS but do not produce the HS-degrading endoglycosidase (Hennes *et al.*, in press). It may be even more likely that both properties – increased secretion of proteochondroitin sulphate and production of the HS-specific endoglycosidase-influence in an as yet unresolved fashion the selection of ESb-cells to a favoured metastatic capacity.

It has been reported that colon carcinoma cells produce more proteochondroitin sulphate than normal colon tissue (Iozzo & Wight, 1982).

The mere production of proteochondroitin sulphate seems to be a peculiar property albeit not a stringently occurring one of cells derived from the haemopoietic system like the tumour cells studied here. For instance, no HS could be isolated from lymphocytes of patients with chronic lymphocytic leukaemia (Capeau *et al.*, 1978), from lymphocytes of patients with chronic myelogenous leukaemia (Metcalfe *et al.*, 1984) and from cloned granulated lymphocytes with natural killer function and cultured mast cells (Bland *et al.*, 1984). Also, proteoglycans of thymic lymphocytes consist largely of proteochondroitin sulphate with only smaller amounts of proteoheparan sulphate. During *in vitro* stimulation of thymic lymphocytes the proportion of both cell-associated and secreted proteochondroitin sulphate was found to be even increased (Hart, 1982).

Recently, it became more evident that proteoglycans produced by cells of the immune system like lymphocytes may not only have a different structure and distribution but also may have different biological functions compared to those of other tissues like epithelium, endothelium or cartilage. Especially, proteochondroitin sulphates seem to be involved in immune processes. For example, a proteochondroitin sulphate localized in granules of natural killer cells is specifically exocytosed when these cells lyse susceptible tumour cell targets (MacDermott *et al.*, 1985). Furthermore, it has been discovered that a 31 kD glycoprotein termed invariant chain (Ii) which is associated with class II antigens of the major histocompatibility complex (MHC) is also the core protein of an MHC class II-associated proteochondroitin sulphate (Sant *et al.*, 1985).

In view of these findings it is now tempting to speculate how increased secretion of proteochondroitin sulphate by tumour cells may influence the metastatic process by taking advantage of cell mediated immune mechanisms. For instance, Spooncer *et al.* (1983) reported that haemopoietically active long-term bone marrow cultures treated with $\beta$-D-xyloside increase their synthesis and secretion of proteochondroitin sulphate but not that of proteoheparan sulphate. This stimulation is matched by a preferential increase in granulocyte-macrophage progenitors (GM-CFC) and in mature granulocytes. We found that the highly metastatic cell line ESb and a non-related highly metastatic mouse tumour, MDAY-D2, produced constitutively more colony stimulating activity (predominantly granulocyte CSA) than the low metastatic cell line Eb which correlated with an increased tendency to granulocytosis in mice bearing the respective tumours (Schwartz & Monner, 1986). The increased stimulation of granulocyte production, possibly influenced by secretion of proteochondroitin sulphate may cause disturbances in haemotopoiesis which could give an advantage for the tumour cells to survive in the host outside the primary tumour.

At this point we have no information about the mechanism of proteoglycan secretion by the tumour cells described here. It may well be that proteoglycans are also included in extracellular plasma membrane vesicles shedded in an increased fashion by ESb compared with Eb cells (Barz et al., 1985).

When comparing metastatic behaviour and expression of proteoglycans in the Eb/ESb system it has to be considered that cell lines ESb-MP grows adherently while cell lines Eb and ESb grow in suspension. Due to this difference proteoglycans may be organized in a different way at the cell surface of ESb-MP-cells which could influence cell-substrate and cell–cell adhesion. For instance, adherently growing melanoma cells restrictively express a proteoglycan on microspikes, a specific microdomain of the cell surface (Garrigues et al., 1986) which is involved in cell-attachment. Furthermore, we do not know yet how proteoglycans of the Eb/ESb system are related to each other and if different species of proteoglycans are produced for cellular expression and secretion. These questions can only be solved by analyzing the complex nature of the respective proteoglycans. The fine biochemical structural analysis of the proteoglycans of the Eb/ESb system is in preparation at present.

### References

ALTEVOGT, P., KURNICK, J.T., KIMURA, A.K., BOSSLET, K. & SCHIRRMACHER, V. (1982). Different expression of Lyt differentiation antigens on cell surface glycoproteins by a murine T-lymphoma line and its high metastatic variant. *Eur. J. Immunol.*, **12**, 300.

BARZ, D., GOPPELT, M., SZAMEL, M., SCHIRRMACHER, V. & RESCH, K. (1985). Characterization of cellular and extracellular plasma membrane vesicles from a non-metastasizing lymphoma (Eb) and its metastasizing variant (ESb). *Biochim. Biophys. Acta*, **814**, 77.

BENKE, R., LANG, E., KOMITOWSKI, D., MUTO, S. & SCHIRRMACHER, V. (1988). Changes in tumour cell adhesives affecting speed of dissemination into blood and internal organs and mode of metastatic growth. *Invas. Metast.*, **8**, 159.

BLAND, K.E., ROSENTHAL, K.L., PLUZNIK, D.H. & 4 others (1984). Glycosaminoglycan profiles in cloned granulated lymphocytes with natural killer function and in cultured mast cells: their potential use as biochemical markers. *J. Immunol.*, **132**, 1937.

CAPEAU, J., PICARD, J. & PAUL-GARDAIS, A. (1978). Isolation of glycosaminoglycans from lymphocytes in chronic lymphoid leukemia. *Biomedicine*, **28**, 124.

COLE, G.J., SCHUBERT, D. & GLASER, L. (1985). Cell-substratum adhesion in chick neural retina depends upon protein-heparan sulphate interactions. *J. Cell Biol.*, **100**, 1192.

FOGEL, M., ALTEVOGT, P. & SCHIRRMACHER, V. (1983). Metastatic potential severely altered by changes in tumour cell adhesiveness and cell surface sialylation. *J. Exp. Med.*, **157**, 371.

FRANSSON, L.-A., SJOBERG, I & CHIARUGI, V.P. (1981). Co-polymeric glycosaminoglycans in transformed cells. Transformation-dependent changes in the self-associating properties of cell-surface heparinsulphate. *J. Biol. Chem.*, **256**, 13044.

FRANSSON, L.-A., CARLSTEDT, L., CÖSTER, L. & MALMSTRÖM, A. (1984). Binding of transferrin to the core protein of fibroblast proteoheparan sulphate. *Proc. Natl Acad. Sci. USA*, **81**, 5657.

FRITZE, L.M.S., REILLY, C.F. & ROSENBERG, R.D. (1985). An antiproliferative heparan sulphate species produced by post confluent smooth muscle cells. *J. Cell Biol.*, **100**, 1041.

GARRIGUES, H.J., LARK, M.W., LARA, S.W., HELLSTRÖM, I., HELLSTRÖM, K.E. & WIGHT, T.N. (1986). The melanoma proteoglycan: restricted expression on microspikes, a specific microdomain of the cell surface. *J. Cell Biol.*, **103**, 1699.

GREILING, H., STUHLSATZ, H.W. & TILLMANNS, U. (1984). Chondroitin, chondroitin 4-sulphate, chondroitin 6-sulphate, and dermatan sulphate. In *Methods of Enzymatic Analysis*, Bergmeyer, H.U. (ed) p. 60, 3rd edition, vol. 6. Verlag Chemie, Weinheim, FRG.

GRIESMACHER, A., HENNES, R., KELLER, R. & GREILING, H. (1987). Proteoglycans from human umbilical vein endothelial cells. *Eur. J. Biochem.*, **168**, 95.

HART, G.W. (1982). Biosynthesis of glycosaminoglycans by thymic lymphocytes. Effects of mitogenic activation. *Biochemistry* **24**, 6088.

HENNES, R., FRANTZEN, F., KELLER, R., SCHIRRMACHER, V. & SCHWARTZ, R. Matrix heparan sulphate but not endothelial cell surface heparan sulphate is degraded by highly metastatic mouse lymphoma cells. *Br. J. Cancer* (In press).

HOLLMANN, J., THIEL, J., SCHMIDT, A. & BUDDECKE, E. (1986). Increased activity of chondroitin sulphate-synthesizing enzymes during proliferation of arterial smooth muscle cells. *Exp. Cell Res.*, **167**, 484.

IOZZO, R.V. (1985). Biology of disease. Proteoglycans: Structure, function and role in neoplasia. *Lab. Invest.*, **53**, 373.

IOZZO, R.V. & WIGHT, T.N. (1982). Isolation and characterization of proteoglycans synthesized by human colon and colon carcinoma. *J. Biol. Chem.*, **257**, 11135.

KELLER, K.L., KELLER, J.M. & MOY, J.N. (1980). Heparan sulphates from Swiss Mouse 3T3 and SV3T3 cells: O-sulphate difference. *Biochemistry*, **19**, 2529.

KELLER, R., SILBERT E.J., FURTHMAYR, H. & MADRI, J.A. (1987). Aortic endothelial cell proteoheparan sulfate. I. Isolation and characterization of plasmamembrane-associated and extracellular species. *Amer. J. Pathol.* **128**, 286.

KUCHERLAPATI, R.S., HILWIG, I., GROPP, A. & RUDDLE, F.H. (1975). Mammalian chromosome identification in interspecific hybrid cells using 'Hoechst 33258'. *Humangenetik*, **27**, 9.

LANDNER, A.D., FUJI, D.K., GOSPODAROWICZ, D. & REICHARDT, L.F. (1982). Characterization of a factor that promotes neurite outgrowth: evidence linking activity to a heparan sulphate proteoglycan. *J. Cell Biol.*, **94**, 574.

LATERRA, J., SILBERT, J.E. & CULP, L.A. (1983). Cell surface heparan sulphate mediates some adhesive responses to glycosamninoglycan-binding matrices, including fibronectin. *J. Cell Biol.* **96**, 112.

LEVITT, D. & HO, P.-L. (1983). Induction of chondroitin sulphate proteoglycan synthesis and secretion in lymphocytes and monocytes. *J. Cell Biol.*, **97**, 351.

MACDERMOTT, R.P., SCHMIDT, R.E., CAULFIELD, J.P. & 6 others (1985). Proteoglycans in cell-mediated cytotoxicity. Identification, localization, and exocytosis of a chondroitin sulphate proteoglycan from human cloned natural killer cells during target cell lysis. *J. Exp. Med.*, **162**, 1771.

METCALFE, D.D., BLAND, C.E. & WASSERMAN, S.I. (1984). Biochemical and functional characterization of proteoglycans isolated from basophils of patients with chronic myelogenous leukemia. *J. Immunol.*, **132**, 1943.

ROBINSON, J., VITI, M. & HÖÖK, M. (1984). Structure and properties of an under-sulphated heparan sulphate proteoglycan synthesized by a rat hepatoma cell line. *J. Cell Biol.*, **98**, 946.

SANT, A.J., CULLEN, S.E., GIACOLETTO, K.S. & SCHWARTZ, B.D. (1985). Invariant chain is the core protein of the Ia-associated chondroitin sulphate proteoglycan. *J. Exp. Med.*, **162**, 1916.

SCHIRRMACHER, V., BOSSLET, K., SHANTZ, G., CLAUER & HÜBSCH, D. (1979*a*). Tumour metastases and cell-mediated immunity in a model system in DBA/2 mice. IV. Antigenic differences between a metastasizing variant and the parental tumour line revealed by cytotoxic T lymphocytes. *Int. J. Cancer*, **23**, 245.

SCHIRRMACHER, V., SHANTZ, G., CLAUER, K., KOMITOWSKI, D., ZIMMERMANN, H.-P. & LOHMANN-MATTHES, M.L. (1979*b*). Tumour metastases and cell-mediated immunity in a model system in DBA/2 mice. I. Tumour invasiveness *in vitro* and metastasis formation *in vivo*. *Int. J. Cancer*, **23**, 233.

SCHWARTZ, R., SCHIRRMACHER, V. & MÜHLRADT, P.F. (1984). Glycoconjugates of murine tumour lines with different metastatic capacities. I. Differences in fucose utlization and in glycoprotein patterns. *Int. J. Cancer*, **33**, 503.

SCHWARTZ, R., KNIEP, B., MÜTHING, J. & MÜHLRADT, P.F. (1985). Glycoconjugates of murine tumour lines with different metastatic capacities. II Diversity of glycolipid composition. *Int. J. Cancer*, **36**, 601.

SCHWARTZ, R. & MONNER, D.A. (1986). Constitutive production of colony-stimulating factor by mouse lymphoma cell lines is correlated with granulocytosis *in vivo*. *Exp. Hematol.*, **14**, 615.

SILVESTRI, L., BAKER, J.R., RODEN, L. & STROUD, R.M. (1981). The $C_{1q}$ inhibitor in serum is a chondroitin 4-sulphate proteoglycan. *J. Biol. Chem.*, **256**, 7383.

SPIRO, R.G. & PARTHASARATHY, N. (1982). Studies on the proteoglycan of basement membranes. In *New trends in basement membrane research* Kuehn, *et al.* (eds) p. 87, Raven Press.

SPOONCER, E., GALLAGHER, J.T., KRIZSA, F. & DEXTER, T.M. (1983). Regulation of haemopoiesis in long-term bone marrow cultures IV. Glycosaminoglycan synthesis and the stimulation of haemopoiesis by $\beta$-D-xylosides. *J. Cell Biol.* **96**, 510.

STAMATOGLOU, S.C. & KELLER, J.M. (1983). Correlation between cell substrate attachment *in vitro* and cell surface heparan sulphate affinity for fibronectin and collagen. *J. Cell Biol.*, **96**, 1820.

STUHLSATZ, H.W., HIRTZEL, F., KELLER, R., COSMA, S. & GREILING, H. (1981). Studies on the polydispersity and heterogeneity of proteokeratan sulphate from calf and porcine cornea. *Hoppe-Seyler's Z. Physiol. Chem.*, **362**, 841.

UNDERHILL, C.B. & KELLER, J.M. (1975). A transformation-dependent difference in heparan sulphate associated with the cell surface. *Biochem. Biophys. Res. Com.*, **63**, 448.

VLODAVSKY, I., FUKS, Z., BAR-NER, M., ARIAV, Y. & SCHIRRMACHER, V. (1983). Lymphoma cell-mediated degradation of sulphated proteoglycans in the subendothelial extracellular matrix: relationship to tumour cell metastasis. *Cancer Res.*, **43**, 2704.

WINTERBORNE, D.J. & MORA, P.T. (1981). Cells selected for high tumourigenicity or transformed by Simian Virus 40 synthesize heparan sulphate with reduced degree of sulphation. *J. Biol. Chem.*, **256**, 4310.